# Longevity and mortality in Kennel Club registered dog breeds in the UK in 2014

**DOI:** 10.1186/s40575-018-0066-8

**Published:** 2018-10-17

**Authors:** T. W. Lewis, B. M. Wiles, A. M. Llewellyn-Zaidi, K. M. Evans, D. G. O’Neill

**Affiliations:** 1 The Kennel Club, Clarges Street, London, W1J 8AB UK; 2 School of Veterinary Medicine and Science, The University of Nottingham, Sutton Bonington Campus, Sutton Bonington, Loughborough, LE12 5RD UK; 3 International Partnership for Dogs, 504547 Grey Rd 1, Georgia Bluffs, ON Canada; 4 Pathobiology and Population Health, The Royal Veterinary College, Hawkshead Lane, North Mymms, Hatfield, AL9 7TA UK

**Keywords:** Pedigree, Lifespan, Purebred, Death, Healthspan, Predisposition, Mortality, Longevity

## Abstract

**Background:**

The domestic dog is one of the most diverse mammalian species, exhibiting wide variations in morphology, behaviour and morbidity across breeds. Therefore, it is not unexpected that breeds should also exhibit variation in mortality and longevity. While shorter longevity per se may not necessarily be a welfare issue, a generally foreshortened lifespan in a breed that is accompanied by a high prevalence of a particular cause of death may reveal potentially serious welfare concerns and highlight scope to improve breed welfare. Survey data gathered directly from owners offer useful insights into canine longevity and mortality that can support the overall evidence base for welfare reforms within breeds.

**Results:**

Mortality data on 5663 deceased dogs registered with the UK Kennel Club were collected from an owner-based survey. The most commonly reported causes of death were old age (13.8%), unspecified cancer (8.7%) and heart failure (4.9%); with 5.1% of deaths reported as unknown cause. Overall median age at death was 10.33 years (interquartile range: 7.17–12.83 years). Breeds varied widely in median longevity overall from the West Highland Terrier (12.71 years) to the Dobermann Pinscher (7.67 years). There was also wide variation in the prevalence of some common causes of death among breeds, and in median longevity across the causes of death.

**Conclusion:**

Substantial variation in the median lifespan and the prominent causes of death exists across breeds. This study has identified some breeds with both a low median lifespan and also a high proportional mortality for one or more specific causes of death that should be considered as both potential welfare concerns as well as opportunities for improvement.

**Electronic supplementary material:**

The online version of this article (10.1186/s40575-018-0066-8) contains supplementary material, which is available to authorized users.

## Plain English Summary

The domestic dog is one of the most diverse mammalian species, exhibiting wide variations in size and shape, common diseases, and behaviours across breeds. Therefore, it is not unexpected that breeds should also exhibit differences both in how long they generally live and their common causes of death. Shorter life alone may not necessarily be viewed as a particular problem; for example, a generally healthy life followed by a fairly rapid decline might be considered a better outcome for the dog than a longer life but with a long and painful decline preceding death. However, an excessively short life in a breed that commonly dies from a particular cause of death may reveal potentially serious welfare concerns. Survey data gathered directly from owners are a useful means to explore longevity and mortality in dogs.

This study collected information on 5663 deaths of dogs registered with the UK Kennel Club from an owner survey. The most common causes of death were old age (13.8%), unspecified cancer (8.7%) and heart failure (4.9%); with 5.1% deaths reported as unknown cause. The overall median average lifespan was 10 years and 4 months but this varied widely across breeds from the West Highland Terrier (12 years 8.5 months) to the Dobermann Pinscher (7 years 8 months). Breeds also differed in their most common causes of death, suggesting that some breeds are susceptible to particular causes of death. There was also variation in the median age at death from the most common causes of death. This information is important because diseases associated with death in younger dogs can be seen as depriving more years of potential life and therefore be considered as having higher welfare impacts.

This study identified substantial variation in lifespan and the prominent causes of death across breeds. Awareness of expected lifespans across breeds may help owners to prepare for the eventual loss of their dog and even assist decision-making when selecting a breed in the first place. Breeds with short lifespans that commonly die of one or more specific causes of death were identified, and highlighted as potential welfare concerns.

## Background

The domestic dog (*Canis familiaris*) is the most phenotypically diverse mammalian species [1]. Wide variations in behaviour [2, 3] and morbidity [4–6] have been reported between breeds. It is therefore not unexpected that variation in longevity should also exist across breeds, and there is some prior evidence to this effect. The median longevity of breeds under first opinion veterinary care in the UK ranged from 14.2 years in the Miniature Poodle to 5.5 years in the Dogue de Bordeaux [7] while breeds under referral care in the US ranged from 9.3 years for the Miniature Poodle to 3.5 years for the Rottweiler [8]. Surveys of owners of Kennel Club registered dogs reported median longevity ranges from 15.5 in the Lakeland Terrier to 3.8 in the Dogue de Bordeaux in the UK [9] and from 12.0 in the Shetland Sheepdog to 7.0 in the Bernese Mountain Dog in Denmark [10]. Accurate, up-to-date and representative data on canine welfare-related parameters including longevity are needed to support evidence-based efforts to understand and improve breed-related health and welfare in dogs [11]. Therefore, it is timely to report a more contemporaneous evaluation of longevity in UK Kennel Club registered dogs.

In addition to longevity, accurate information on the common causes of death in dogs (i.e. mortality) can assist prioritisation of disease-specific reforms that may extend life or improve welfare by palliating the dying process or facilitating earlier diagnosis [7]. A previous survey of pedigree dog owners in the UK identified cancer (27%), ‘old age’ (18%) and cardiac conditions (11%) as the most common causes of death [9]. The most common causes of death in dogs under first opinion veterinary care in the UK were neoplastic diseases (16.5%), musculoskeletal disorders (11.3%) and neurological disorders (11.2%) [7], while the most common causes of death in kennel club registered dogs in Denmark were ‘old age’ (20.4%), cancer (14.5%) and behaviour problems (6.4%) [10]. These studies highlight some common trends that would benefit from more recent data relating to the current UK population of pedigree dogs. Furthermore, just as a generally shorter lifespan of some breeds compared to others has been reported [7, 9], there is also some evidence from single-breed studies that proportional mortality from various causes of death varies across breeds [12–14] and this would benefit from exploration in a multi-breed study to compare the common causes of death across breeds.

Much of the available evidence on longevity and mortality of dogs is based on veterinary or insurance data that was not originally recorded for research purposes [7]. Primary-care practice data closely represent the general dog population, including a broad range of causes of death and contemporaneous recording at the point of death by the veterinarian to reduce the effects of recall bias [15]. However, limited clinical work-up may lead to some disease misclassification and there are also substantial technical complexities related to the acquisition, management and analysis of large primary-care practice datasets [15]. Pet insurance data have also been used for longevity and mortality research [16–18] but insurance data are limited by biases from owner demographics, older animals often becoming uninsured, excluded conditions, threshold financial excesses for claims and age-limited life cover [19, 20]. Veterinary clinical data and insurance data cover both Kennel Club registered (i.e. pedigree) and non-Kennel Club registered purebred dogs that cannot generally be distinguished and analysed separately within these datasets. Therefore, inference for owners of pedigree dogs from results based on these data resources may be confounded by beliefs about differing health status between the registered and non-registered subsets of purebred dogs. Data collected specifically on Kennel Club registered dogs would allow results that pertain directly to the pedigreed segment of the overall dog population.

Longevity reflects the period between the date of birth and the date of death [7]. Data on the date of birth is routinely recorded by breed and kennel club registries close to the time of birth and also by veterinary practices and insurance companies at variable time points in a dog’s life [7, 10, 17]. Unfortunately, the date and cause of death in dogs is less commonly reported to, or recorded by, breed registries, insurance companies or often even veterinary practices and therefore it has historically been challenging to access longevity and mortality data for large and representative cohorts of dogs from secondary data sources [7, 20]. An alternative research option is to institute a primary data collection process that collects information on the cause of, and age at, death of dogs directly from owners. This approach was successfully used in 2004 when the Kennel Club/British Small Animal Veterinary Association (BSAVA) Committee with the Epidemiology Unit at the Animal Health Trust carried out a nationwide direct survey of UK pedigree dog owners to identify the most common causes of death in UK dog breeds [9]. This approach offers relatively large volumes of survival data on breeds that are known to be pedigree but also accepts the well-recognised shortcomings to owner surveys, including variable response rates, recall and selection biases and difficulties in validation that all require strong study planning to limit or understand their impacts [15]. In the end, results from well-designed owner surveys can add unique insights that can complement other data sources to build more accurate overall representations [15].

This paper reports the results of longevity and mortality analyses from a large survey of owners of Kennel Club registered dogs in the UK carried out in 2014. The objectives of the study were to report the longevity and the most common causes of death both overall and within-breed from Kennel Club registered pedigree dogs that died over the preceding 10 years. The study aimed to add to the extant body of evidence on canine longevity and mortality and provide a reliable evidence base to support welfare reforms within breeds.

## Methods

### Survey

A mortality survey on previously-owned Kennel Club registered dogs was undertaken in conjunction with a larger survey of owners of living pedigree dogs registered with the Kennel Club. The methods and results of this larger survey have previously been reported [4]. In brief, the surveys were applied online using a web-based survey tool (SurveyMonkey) and were open from 8th November 2014 until 31st December 2014. There were 546,836 invitations emailed to owners of Kennel Club registered dogs. The surveys were also promoted on social media (Twitter and Facebook), the Kennel Club website, in the dog press (Dog World and Our Dogs) and within breeds by personal communication from Breed Health Coordinators. Participants in the mortality survey were asked to provide details on Kennel Club registered dogs they had owned and which had died during the 10-year period prior to the survey (1st January 2005 to 25th December 2014) with no stipulations made on date of birth. Dog owners were able to participate in either the mortality survey or the morbidity survey, or both, but dogs for which participants provided mortality information were necessarily different to those still living for which they may have provided morbidity information. The section of the questionnaire relevant to mortality contained nine additional questions, including the identity of deceased dog(s) (Kennel Club name and number), date of death, cause(s) of death (recordable in free-text format), whether death was unassisted or euthanasia, and whether a post mortem was performed.

### Data processing

The online survey closed on 31st December 2014 and the data were exported from SurveyMonkey to a spreadsheet in Microsoft Excel CSV format for cleaning and verification against the Kennel Club database in Microsoft Access (Microsoft Corporation, 2017). Each dog was linked to the Kennel Club database via its unique Kennel Club number to verify the information provided in the survey and to extract additional demographic data including the date of birth. Only dogs with a verified Kennel Club registration were included in the analyses. Data on individual dogs were anonymised prior to analysis. Age at death was calculated as the difference between the recorded date of birth on the Kennel Club database and the date of death provided by the owner.

Each dog had a single primary cause of death extracted from the survey free text that was mapped to a standardised disorder list developed from the VeNom coding system [21]. For dogs recorded with multiple causes of death in the free-text and where no primary cause was identifiable, the first stated cause of death was assigned. ‘Old age’ was assigned when either “age” or “old age” was stated as the sole cause of death. ‘Old age combinations’ was assigned as cause of death when “age” or “old age” was stated together with free text indicative of frailty and decline, such as arthritis, incontinence, heart failure, progressive loss of mobility / collapsing / hind legs failing, failing appetite etc. Dogs with senility, dementia or cognitive dysfunction stated as the sole cause of death were coded as ‘senile dementia/cognitive dysfunction’. A category called ‘unknown’ was used when the stated cause of death could not be confidently determined or when the words “unknown,” “undiagnosed” or “died” were used.

### Statistical analysis

Data analyses used R (an online open-access language and environment for statistical computing and graphics) [22] and Matlab [23]. Proportional mortality estimates for specific causes of death were calculated by dividing the number of deaths from that cause by the total number of deaths in the same cohort. The Wilson approximation method was used to calculate 95% confidence intervals [24]:

$$ \frac{\left(2 np+{z}^2\pm z\sqrt{\left({z}^2+4 npq\right)}\right)}{2\left(n+{z}^2\right)} $$

where *n* is the number of reported deaths, *p* is the reported incidence of the cause of death in question, *q* is the deaths due to a separate cause (*1-p*), and *z* is the 1-α/2 point of the standard Normal distribution (1.96 for 95% confidence interval [95% CI]).

The ‘overall proportional mortality’ (OPM) estimates for specific causes of death were reported based on all dogs included in the survey. The ‘within breed proportional mortality’ (WBPM) for the subset of causes of death with ≥50 deaths ascribed in the overall study (*n* = 25 causes) was reported for those breeds with ≥50 unique deaths reported (n = 25 breeds). Differences between OPM and WBPM (n = 25 breeds) were assessed for each of the 25 common causes of death using the chi-squared test with Holm adjusted *P*-values to account for multiple testing [25].

The median age at death was reported overall and for each of the 25 breeds with ≥50 unique deaths, to mitigate the disproportionate effects excessively large and small values have on the mean [26]. The median age at death was also reported for each of the 25 common causes of death (with ≥50 reports overall). The approximate 95% CI of the median, as used to depict the ‘notch’ on the Box and Whisker plot, was calculated as:

$$ \frac{1.57\times IQR}{\sqrt{n}} $$

where *IQR* is the interquartile range and *n* is the number of responses [27].

## Results

The mortality survey collected responses from 4287 owners representing 5663 deceased dogs across 179 breeds (82.3% of the 215 breeds recognised by the Kennel Club^1^). The median count of deaths per breed was 11 (IQR: 4–28.5 range 1–728, full list reported in Additional file 1). Twenty-five breeds had ≥50 reported deaths (Labrador Retriever, Golden Retriever, German Shepherd Dog, Cocker Spaniel, Flat Coated Retriever, English Springer Spaniel, Cavalier King Charles Spaniel, Boxer, Border Collie, Dobermann, Border Terrier, Irish Setter, West Highland White Terrier, Bernese Mountain Dog, Miniature Schnauzer, Rottweiler, Weimaraner, Staffordshire Bull Terrier, Shetland Sheepdog, Whippet, Gordon Setter, Newfoundland, Bearded Collie, Dalmatian and Pointer) and were included as individual breeds in the analyses.

### Longevity

**Fig. 1:**
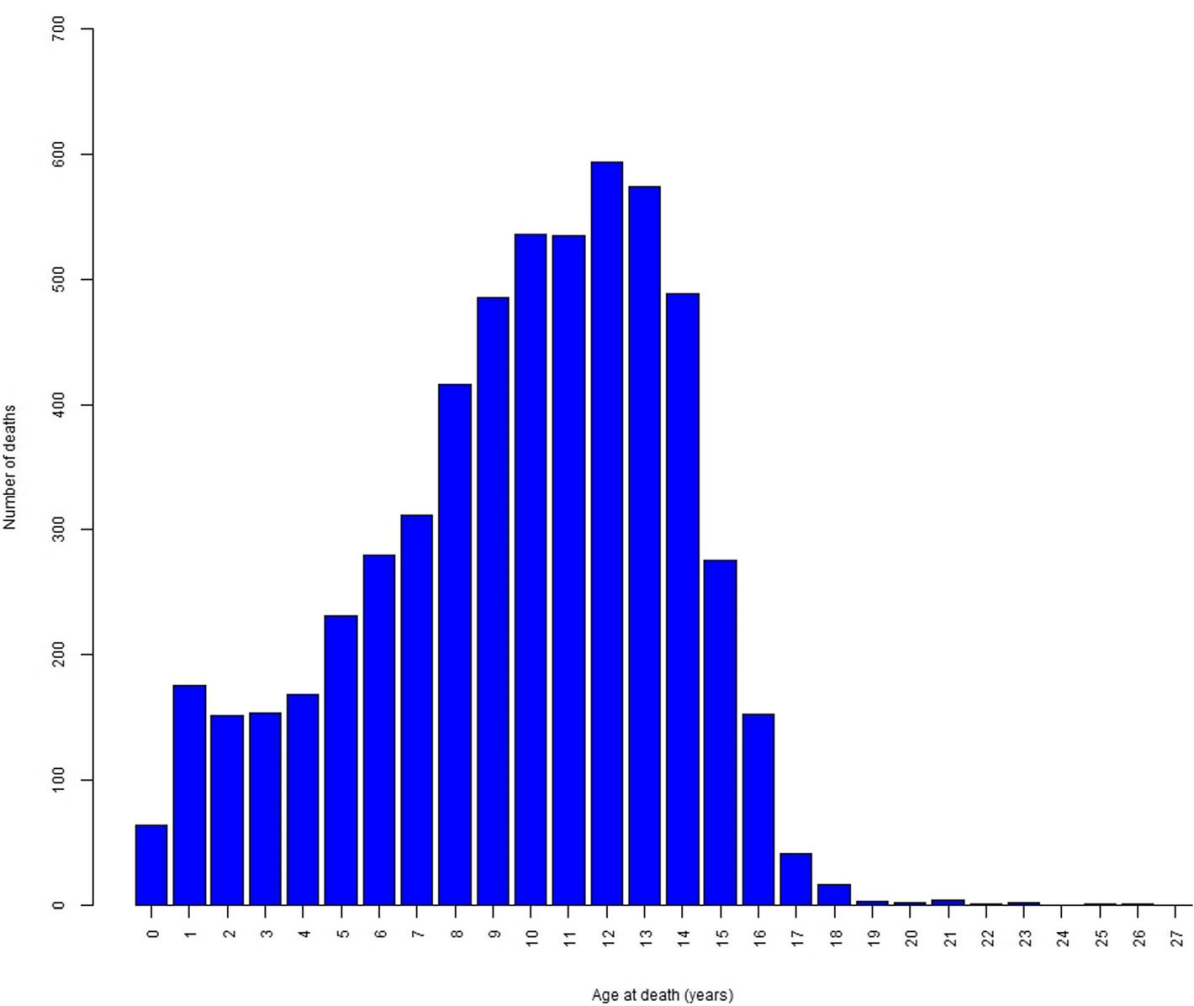
Distribution of age at death from all dogs in the survey. The distribution of age at death (in years) from all dogs in the survey (*n* = 5663)

The overall median age at death across all breeds was 124 months [10.33 years] (95% CI: 122.57 to 125.43 months, IQR: 86–154 months, range: 0–317 months). The distribution of age at death across all breeds is shown in Fig. 1. Of all deaths reported, 79.58% involved euthanasia and 5.56% had a post-mortem.

**Fig. 2:**
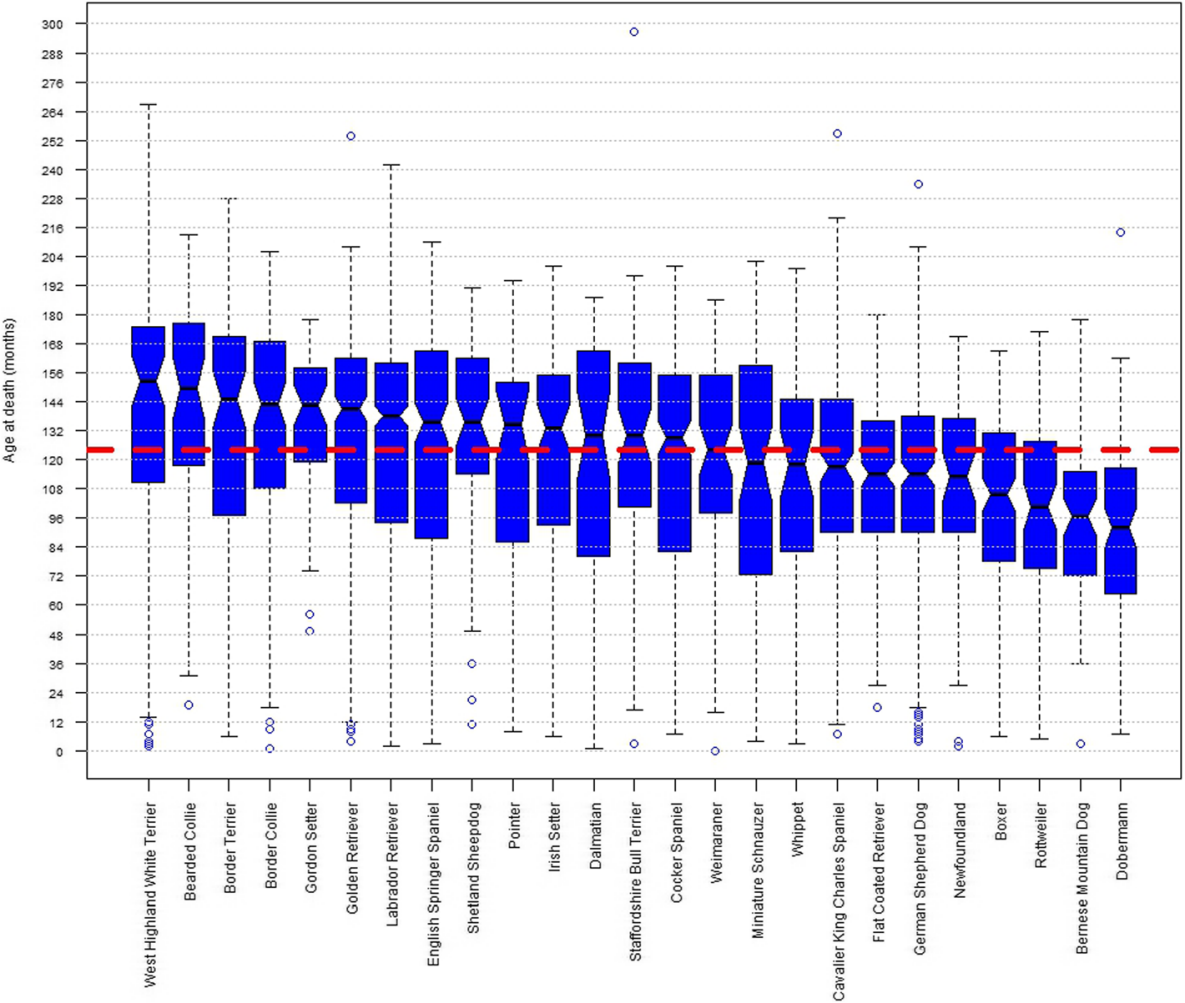
Box and Whisker plot of age at death across breeds. Notched Box and Whisker plot of age at death (months) across breeds, for the 25 breeds with ≥50 deaths reported. ‘Notches’ in the boxes indicate the approximate 95% confidence interval of the median. The red dashed line indicates the median age at death of all dogs in the survey (n = 5663) of 124 months, and the thickness of this line approximates to the 95% confidence interval (122.57 to 125.43 months)

The median longevity varied widely across the 25 breeds with ≥50 deaths reported and ranged from 152.5 months [12.71 years] in the West Highland Terrier to 92 months [7.67 years] in the Dobermann Pinscher (Table 1). The upper boundary of the approximate 95% confidence interval of median age of death was lower than 122.57 months (the lower boundary of the approximate 95% confidence interval of the median using all data) in six of the twenty-five breeds (Flat Coated Retriever, German Shepherd Dog, Boxer, Rottweiler, Bernese Mountain Dog and Dobermann) highlighting these as shorter lived breeds. The lower boundary of the approximate 95% confidence interval of median age at death was higher than 125.43 months (the upper boundary of the approximate 95% confidence interval of the median using all data) in eight of the twenty-five breeds (West Highland White Terrier, Bearded Collie, Border Terrier, Border Collie, Gordon Setter, Golden Retriever, Labrador Retriever, English Springer Spaniel) highlighting these as longer lived breeds. A notched box and whisker plot (Fig. 2) shows variations in the distribution of age at death within breeds, for the 25 breeds with ≥50 deaths reported (‘notches’ in the boxes indicate the approximate 95% confidence interval of the median).

**Table 1:** Age at death statistics of the 25 breeds (with > 50 reported deaths), including the number of deaths reported (N), the contribution per breed (as a percentage of all deaths reported in the survey), the median, inter-quartile range (IQR), range (minimum and maximum) and 95% confidence interval of the median of age of death in months

Breed	No. Deaths (N)	% of all deaths	Parameters of age at death (months) by breed (with *n* ≥ 50)			
			Median	IQR	Range	95% CI
Labrador Retriever	728	12.86	138	94–160	2–242	3.86
Golden Retriever	373	6.59	141	102–162	4–254	4.91
German Shepherd Dog	279	4.93	114	90–138	4–234	4.54
Cocker Spaniel	266	4.70	129	82–155	7–200	7.07
Flat Coated Retriever	225	3.97	114	90–136	18–180	4.85
English Springer Spaniel	224	3.96	135.5	87.5–165	3–210	8.18
Cavalier King Charles Spaniel	222	3.92	117	90–145	7–255	5.83
Boxer	170	3.00	105.5	78–131	6–165	6.42
Border Collie	119	2.10	143	107.5–169	1–206	8.91
Dobermann	99	1.75	92	63.75–117.25	7–214	8.50
Border Terrier	98	1.73	145	97–171	6–228	11.81
Irish Setter	98	1.73	133	93–155	6–200	9.90
West Highland White Terrier	96	1.70	152.5	110.5–175	2–267	10.40
Bernese Mountain Dog	78	1.38	96.5	72–115	3–178	7.69
Miniature Schnauzer	76	1.34	118.5	72.5–159	4–202	15.68
Rottweiler	76	1.34	100.5	75–127.5	5–173	9.52
Weimaraner	74	1.31	124	98–155	0–186	10.47
Staffordshire Bull Terrier	71	1.25	130	100.25–160.5	3–297	11.30
Shetland Sheepdog	56	0.99	135.5	114–162	11–191	10.14
Whippet	55	0.97	118	81.5–146	3–199	13.74
Gordon Setter	54	0.95	142.5	119–158	49–178	8.39
Newfoundland	53	0.94	113	90–137	2–171	10.20
Bearded Collie	52	0.92	149.5	117.5–176.5	19–213	12.93
Dalmatian	52	0.92	130	80–165	1–187	18.62
Pointer	50	0.88	134.5	86–152	8–194	14.75
Cumulative total	3744	66.11		–	–	
All responses from all breeds	5663	100%	124	86–154	0–317	1.43

### Mortality

There were 206 unique causes of death reported across the 5663 deceased dogs. The most frequently reported specific causes of death were ‘old age’ (*n* = 780, OPM = 13.77%), ‘cancer – unspecified’ (*n* = 492, OPM = 8.69%) and ‘heart failure’ (*n* = 277, OPM = 4.89%). There were also 290 dogs recorded with ‘unknown’ cause of death (OPM = 5.12%). The total number of reports and overall proportional mortality (with median, IQR and range, and 95% confidence intervals) of the 30 most common causes of death are listed in Table 2 (the full list is reported in Additional file 1).

**Table 2:** Age at death statistics from the 30 most commonly reported causes, including the number of deaths reported (N), the contribution per cause (as a percentage of all deaths reported in the survey), the median, inter-quartile range (IQR), range (minimum and maximum) and 95% confidence interval of the median of age of death in months

Disease	No. Deaths (N)	Prevalence (%)	Parameters of age at death (months) by breed (with n ≥ 50)			
			Median	IQR	Range	95% CI
Old Age	780	13.77	164.5	148–177	69–267	1.64
Cancer - unspecified	492	8.69	123	98.5–145	2–255	3.31
Unknown	290	5.12	112	71–147	0–241	7.05
Heart Failure	277	4.89	119	90.75–147	0–207	5.34
Kidney Failure	239	4.22	125	85–152	3–210	6.85
Old Age combinations	229	4.04	155	139–169	86–216	3.13
Bone tumour	192	3.39	110.5	85–132.5	18–242	5.42
Lymphoma	180	3.18	95.5	61–126	3–171	7.65
Cardiomyopathy	166	2.93	114	82–142	1–220	7.36
Brain tumour	159	2.81	103	73.5–136	0–195	7.83
Stroke	136	2.40	152.5	129–168	7–191	5.28
Splenic tumour	129	2.28	126	101.75–146.75	16–180	6.26
Hepatic liver tumour	115	2.03	119	91–142	20–183	7.51
Road Traffic Accident	102	1.80	38.5	14–74	1–176	9.39
Liver Failure	89	1.57	136	96–152.25	4–195.36	9.42
Epilepsy	88	1.55	68	39–96	4–180	9.60
Gastric dilation-volvulus (Bloat)	87	1.54	101	60–127.75	14–166.68	11.48
Aggression	85	1.50	39	21–75.25	7–157	9.30
Seizure	82	1.45	130.5	81–153	4–192	12.56
Gastric tumour	78	1.38	120.5	95–143	25–198	8.59
Arthritis	77	1.36	151	129.42–168	18–219	6.95
Oral tumour	72	1.27	133.5	103.5–154	27–203	9.40
Lung tumour	55	0.97	117	96–135.5	32–317	8.42
Mammary tumour	53	0.94	126	111–148.25	78–190	8.08
Hyperadrenocorticism (Cushing’s Disease)	50	0.88	138	117–158	69–188	9.16
Intestinal tumour	49	0.87	127	93–149	28–195	12.64
Skin tumour	45	0.79	112	76.75–136	14–179	13.96
Surgical complication	44	0.78	68	31.5–107.5	2–193	18.10
Pancreatitis	42	0.74	109	67–132	13–191	15.85
Leukaemia	40	0.71	94	60.5–114	13–177	13.37
Cumulative total	4522	79.85		–	–	
All responses from all breeds	5663	100%	124	86–154	0–317	1.43

The WBPM (and 95% CI) for the causes of death with ≥50 reports (*n* = 25) are shown for the 25 breeds with ≥50 reports (*n* = 3744 deaths) (Table 3). The WBPM for “old age” ranged from 3.85% in Bernese Mountain Dogs to 25.0% in Bearded Collies. The WBPM for ‘cancer – unspecified’ ranged from 0.00% in Gordon Setters to 19.56% in Flat Coated Retrievers. The WBPM for ‘heart failure’ ranged from 0.00% in Whippets to 19.82% in Cavalier King Charles Spaniels.

**Table 3:** The within breed proportional mortality (WBPM) and 95% confidence intervals (in parentheses) for the 25 most commonly reported causes of death for the 25 breeds with ≥50 reports (*n* = 3744 deaths)

Cause of death	Aggression	Arthritis	Bone tumour	Brain tumour	Cancer-unspecified	Cardiomyopathy	Epilepsy	Gastric dilation-volvulus (Bloat)	Gastric tumour	Heart Failure	Hepatic liver tumour	Hyperadrenocorticism (Cushing’s Disease)	Kidney Failure	Liver Failure	Lung tumour	Lymphoma	Mammary tumour	Old Age	Old Age combinations	Oral tumour	Road Traffic Accident	Seizure	Splenic tumour	Stroke	Unknown
Bearded Collie	0.00 (0.00–6.88)	3.85 (1.06–12.98)	1.92 (0.34–10.12)	5.77 (1.98–15.64)	1.92 (0.34–10.12)	1.92 (0.34–10.12)	1.92 (0.34–10.12)	0.00 (0.00–6.88)	0.00 (0.00–6.88)	1.92 (0.34–10.12)	3.85 (1.06–12.98)	1.92 (0.34–10.12)	1.92 (0.34–10.12)	1.92 (0.34–10.12)	0.00 (0.00–6.88)	3.85 (1.06–12.98)	0.00 (0.00–6.88)	25.00 (15.23–38.21)	9.62 (4.18–20.61)	0.00 (0.00–6.88)	1.92 (0.34–10.12)	0.00 (0.00–6.88)	0.00 (0.00–6.88)	5.77 (1.98–15.64)	3.85 (1.06–12.98)
Bernese Mountain Dog	0.00 (0.00–4.69)	1.28 (0.23–6.91)	7.69 (3.57–15.78)	2.56 (0.71–8.88)	17.95 (11.00–27.90)	0.00 (0.00–4.69)	0.00 (0.00–4.69)	3.85 (1.32–10.71)	1.28 (0.23–6.91)	1.28 (0.23–6.91)	2.56 (0.71–8.88)	0.00 (0.00–4.69)	5.13 (2.01–12.46)	1.28 (0.23–6.91)	3.85 (1.32–10.71)	10.26 (5.29–18.95)	0.00 (0.00–4.69)	3.85 (1.32–10.71)	1.28 (0.23–6.91)	1.28 (0.23–6.91)	1.28 (0.23–6.91)	0.00 (0.00–4.69)	3.85 (1.32–10.71)	2.56 (0.71–8.88)	3.85 (1.32–10.71)
Border Collie	2.52 (0.86–7.15)	2.52 (0.86–7.15)	1.68 (0.46–5.92)	7.56 (4.03–13.75)	6.72 (3.45–12.71)	0.00 (0.00–3.13)	5.04 (2.33–10.56)	0.84 (0.15–4.61)	0.84 (0.15–4.61)	4.20 (1.81–9.46)	2.52 (0.86–7.15)	1.68 (0.46–5.92)	4.20 (1.81–9.46)	0.84 (0.15–4.61)	0.00 (0.00–3.13)	5.04 (2.33–10.56)	1.68 (0.46–5.92)	8.40 (4.63–14.78)	3.36 (1.31–8.32)	0.84 (0.15–4.61)	0.84 (0.15–4.61)	5.04 (2.33–10.56)	1.68 (0.46–5.92)	1.68 (0.46–5.92)	3.36 (1.31–8.32)
Border Terrier	0.00 (0.00–3.77)	1.02 (0.18–5.56)	2.04 (0.56–7.14)	6.12 (2.84–12.72)	4.08 (1.60–10.03)	1.02 (0.18–5.56)	3.06 (1.05–8.62)	1.02 (0.18–5.56)	0.00 (0.00–3.77)	2.04 (0.56–7.14)	1.02 (0.18–5.56)	6.12 (2.84–12.72)	3.06 (1.05–8.62)	5.10 (2.20–11.39)	1.02 (0.18–5.56)	4.08 (1.60–10.03)	1.02 (0.18–5.56)	14.29 (8.70–22.56)	3.06 (1.05–8.62)	0.00 (0.00–3.77)	7.14 (3.50–14.02)	2.04 (0.56–7.14)	2.04 (0.56–7.14)	2.04 (0.56–7.14)	4.08 (1.60–10.03)
Boxer	0.59 (0.10–3.26)	1.18 (0.32–4.19)	2.35 (0.92–5.89)	15.29 (10.66–21.47)	12.35 (8.22–18.15)	4.12 (2.01–8.25)	0.59 (0.10–3.26)	1.76 (0.60–5.06)	1.18 (0.32–4.19)	7.06 (4.08–11.93)	0.59 (0.10–3.26)	0.00 (0.00–2.21)	2.94 (1.26–6.70)	0.00 (0.00–2.21)	0.59 (0.10–3.26)	5.88 (3.23–10.49)	2.94 (1.26–6.70)	4.12 (2.01–8.25)	2.94 (1.26–6.70)	2.35 (0.92–5.89)	1.76 (0.60–5.06)	2.35 (0.92–5.89)	1.18 (0.32–4.19)	1.18 (0.32–4.19)	5.29 (2.81–9.75)
Cavalier King Charles Spaniel	0.00 (0.00–1.70)	0.00 (0.00–1.70)	0.00 (0.00–1.70)	1.35 (0.46–3.90)	1.80 (0.70–4.54)	10.81 (7.37–15.58)	1.80 (0.70–4.54)	0.00 (0.00–1.70)	0.45 (0.08–2.51)	19.82 (15.11–25.56)	0.45 (0.08–2.51)	1.80 (0.70–4.54)	6.76 (4.14–10.85)	1.35 (0.46–3.90)	0.45 (0.08–2.51)	0.90 (0.25–3.22)	0.45 (0.08–2.51)	12.16 (8.49–17.12)	0.45 (0.08–2.51)	0.90 (0.25–3.22)	1.80 (0.70–4.54)	2.70 (1.24–5.77)	0.45 (0.08–2.51)	1.80 (0.70–4.54)	1.80 (0.70–4.54)
Cocker Spaniel	4.14 (2.32–7.25)	0.75 (0.21–2.70)	1.13 (0.38–3.26)	1.50 (0.59–3.80)	10.53 (7.38–14.79)	3.01 (1.53–5.82)	1.13 (0.38–3.26)	0.38 (0.07–2.10)	0.75 (0.21–2.70)	4.51 (2.60–7.72)	1.88 (0.81–4.32)	0.00 (0.00–1.42)	7.89 (5.22–11.77)	2.63 (1.28–5.33)	0.38 (0.07–2.10)	2.26 (1.04–4.83)	0.75 (0.21–2.70)	9.02 (6.14–13.07)	4.14 (2.32–7.25)	1.50 (0.59–3.80)	4.14 (2.32–7.25)	0.38 (0.07–2.10)	0.75 (0.21–2.70)	2.26 (1.04–4.83)	6.77 (4.32–10.44)
Dalmatian	0.00 (0.00–6.88)	1.92 (0.34–10.12)	1.92 (0.34–10.12)	1.92 (0.34–10.12)	7.69 (3.03–18.17)	1.92 (0.34–10.12)	0.00 (0.00–6.88)	1.92 (0.34–10.12)	1.92 (0.34–10.12)	5.77 (1.98–15.64)	0.00 (0.00–6.88)	0.00 (0.00–6.88)	3.85 (1.06–12.98)	0.00 (0.00–6.88)	0.00 (0.00–6.88)	5.77 (1.98–15.64)	0.00 (0.00–6.88)	9.62 (4.18–20.61)	11.54 (5.40–22.97)	0.00 (0.00–6.88)	0.00 (0.00–6.88)	0.00 (0.00–6.88)	0.00 (0.00–6.88)	1.92 (0.34–10.12)	5.77 (1.98–15.64)
Dobermann	3.03 (1.04–8.53)	0.00 (0.00–3.74)	4.04 (1.58–9.93)	3.03 (1.04–8.53)	10.10 (5.58–17.60)	19.19 (12.64–28.04)	1.01 (0.18–5.50)	3.03 (1.04–8.53)	0.00 (0.00–3.74)	9.09 (4.86–16.38)	1.01 (0.18–5.50)	0.00 (0.00–3.74)	1.01 (0.18–5.50)	4.04 (1.58–9.93)	0.00 (0.00–3.74)	8.08 (4.15–15.14)	1.01 (0.18–5.50)	4.04 (1.58–9.93)	1.01 (0.18–5.50)	1.01 (0.18–5.50)	0.00 (0.00–3.74)	0.00 (0.00–3.74)	1.01 (0.18–5.50)	0.00 (0.00–3.74)	4.04 (1.58–9.93)
English Springer Spaniel	4.02 (2.13–7.46)	0.89 (0.25–3.20)	2.23 (0.96–5.12)	3.13 (1.52–6.31)	8.04 (5.14–12.34)	1.34 (0.46–3.86)	2.23 (0.96–5.12)	0.00 (0.00–1.69)	0.89 (0.25–3.20)	3.13 (1.52–6.31)	0.89 (0.25–3.20)	0.00 (0.00–1.69)	4.91 (2.76–8.58)	4.02 (2.13–7.46)	0.45 (0.08–2.49)	3.57 (1.82–6.89)	2.23 (0.96–5.12)	15.18 (11.07–20.46)	4.02 (2.13–7.46)	0.89 (0.25–3.20)	1.34 (0.46–3.86)	0.45 (0.08–2.49)	2.23 (0.96–5.12)	3.57 (1.82–6.89)	6.25 (3.76–10.22)
Flat Coated Retriever	0.00 (0.00–1.68)	1.33 (0.45–3.85)	10.67 (7.27–15.38)	1.78 (0.69–4.48)	19.56 (14.90–25.23)	1.33 (0.45–3.85)	0.00 (0.00–1.68)	2.22 (0.95–5.10)	1.33 (0.45–3.85)	2.67 (1.23–5.69)	2.22 (0.95–5.10)	1.33 (0.45–3.85)	2.67 (1.23–5.69)	0.44 (0.08–2.47)	1.33 (0.45–3.85)	4.00 (2.12–7.43)	0.44 (0.08–2.47)	4.89 (2.75–8.54)	2.67 (1.23–5.69)	1.33 (0.45–3.85)	1.33 (0.45–3.85)	0.44 (0.08–2.47)	8.00 (5.12–12.29)	1.78 (0.69–4.48)	2.22 (0.95–5.10)
German Shepherd Dog	3.23 (1.71–6.02)	1.43 (0.56–3.63)	1.79 (0.77–4.13)	1.43 (0.56–3.63)	7.53 (4.98–11.23)	2.51 (1.22–5.09)	2.15 (0.99–4.61)	3.23 (1.71–6.02)	0.72 (0.20–2.58)	2.15 (0.99–4.61)	2.51 (1.22–5.09)	0.00 (0.00–1.36)	3.23 (1.71–6.02)	0.00 (0.00–1.36)	0.72 (0.20–2.58)	1.43 (0.56–3.63)	1.08 (0.37–3.11)	12.90 (9.47–17.35)	4.66 (2.74–7.81)	0.72 (0.20–2.58)	0.72 (0.20–2.58)	1.08 (0.37–3.11)	4.30 (2.48–7.37)	1.43 (0.56–3.63)	9.32 (6.44–13.30)
Golden Retriever	0.80 (0.27–2.34)	2.95 (1.65–5.20)	3.49 (2.05–5.87)	1.34 (0.57–3.10)	7.24 (5.02–10.33)	1.61 (0.74–3.46)	2.95 (1.65–5.20)	0.54 (0.15–1.93)	1.61 (0.74–3.46)	2.95 (1.65–5.20)	2.14 (1.09–4.17)	0.00 (0.00–1.02)	4.56 (2.86–7.18)	0.80 (0.27–2.34)	1.34 (0.57–3.10)	5.90 (3.93–8.77)	0.27 (0.05–1.50)	19.84 (16.11–24.18)	5.09 (3.28–7.82)	2.14 (1.09–4.17)	2.41 (1.27–4.52)	1.34 (0.57–3.10)	1.88 (0.91–3.82)	4.29 (2.66–6.85)	3.49 (2.05–5.87)
Gordon Setter	3.70 (1.02–12.54)	1.85 (0.33–9.77)	3.70 (1.02–12.54)	1.85 (0.33–9.77)	0.00 (0.00–6.64)	1.85 (0.33–9.77)	0.00 (0.00–6.64)	3.70 (1.02–12.54)	1.85 (0.33–9.77)	1.85 (0.33–9.77)	1.85 (0.33–9.77)	0.00 (0.00–6.64)	3.70 (1.02–12.54)	0.00 (0.00–6.64)	1.85 (0.33–9.77)	1.85 (0.33–9.77)	1.85 (0.33–9.77)	20.37 (11.77–32.90)	3.70 (1.02–12.54)	5.56 (1.91–15.11)	0.00 (0.00–6.64)	3.70 (1.02–12.54)	1.85 (0.33–9.77)	3.70 (1.02–12.54)	5.56 (1.91–15.11)
Irish Setter	0.00 (0.00–3.77)	1.02 (0.18–5.56)	2.04 (0.56–7.14)	2.04 (0.56–7.14)	6.12 (2.84–12.72)	0.00 (0.00–3.77)	4.08 (1.60–10.03)	6.12 (2.84–12.72)	1.02 (0.18–5.56)	8.16 (4.19–15.29)	1.02 (0.18–5.56)	0.00 (0.00–3.77)	2.04 (0.56–7.14)	3.06 (1.05–8.62)	0.00 (0.00–3.77)	2.04 (0.56–7.14)	0.00 (0.00–3.77)	16.33 (10.31–24.89)	4.08 (1.60–10.03)	2.04 (0.56–7.14)	1.02 (0.18–5.56)	2.04 (0.56–7.14)	2.04 (0.56–7.14)	1.02 (0.18–5.56)	6.12 (2.84–12.72)
Labrador Retriever	0.82 (0.38–1.79)	3.43 (2.34–5.02)	2.47 (1.57–3.87)	1.79 (1.05–3.03)	10.71 (8.67–13.17)	1.10 (0.56–2.15)	1.10 (0.56–2.15)	0.55 (0.21–1.40)	1.51 (0.85–2.69)	3.98 (2.79–5.66)	2.20 (1.36–3.54)	0.14 (0.02–0.77)	1.65 (0.95–2.86)	1.92 (1.15–3.20)	1.51 (0.85–2.69)	2.47 (1.57–3.87)	0.69 (0.29–1.60)	20.33 (17.57–23.41)	6.46 (4.89–8.48)	1.92 (1.15–3.20)	2.06 (1.25–3.37)	1.37 (0.75–2.51)	2.61 (1.68–4.04)	1.92 (1.15–3.20)	5.77 (4.30–7.71)
Miniature Schnauzer	0.00 (0.00–4.81)	0.00 (0.00–4.81)	0.00 (0.00–4.81)	3.95 (1.35–10.97)	7.89 (3.67–16.17)	1.32 (0.23–7.08)	3.95 (1.35–10.97)	0.00 (0.00–4.81)	0.00 (0.00–4.81)	2.63 (0.72–9.10)	3.95 (1.35–10.97)	5.26 (2.07–12.77)	5.26 (2.07–12.77)	3.95 (1.35–10.97)	2.63 (0.72–9.10)	5.26 (2.07–12.77)	0.00 (0.00–4.81)	13.16 (7.31–22.55)	2.63 (0.72–9.10)	0.00 (0.00–4.81)	5.26 (2.07–12.77)	2.63 (0.72–9.10)	0.00 (0.00–4.81)	1.32 (0.23–7.08)	6.58 (2.84–14.49)
Newfoundland	0.00 (0.00–6.76)	1.89 (0.33–9.94)	13.21 (6.55–24.84)	0.00 (0.00–6.76)	11.32 (5.29–22.58)	1.89 (0.33–9.94)	0.00 (0.00–6.76)	7.55 (2.97–17.86)	0.00 (0.00–6.76)	7.55 (2.97–17.86)	3.77 (1.04–12.75)	0.00 (0.00–6.76)	1.89 (0.33–9.94)	1.89 (0.33–9.94)	0.00 (0.00–6.76)	0.00 (0.00–6.76)	0.00 (0.00–6.76)	13.21 (6.55–24.84)	9.43 (4.10–20.25)	1.89 (0.33–9.94)	1.89 (0.33–9.94)	0.00 (0.00–6.76)	1.89 (0.33–9.94)	0.00 (0.00–6.76)	7.55 (2.97–17.86)
Pointer	0.00 (0.00–7.14)	2.00 (0.35–10.50)	6.00 (2.06–16.22)	6.00 (2.06–16.22)	8.00 (3.15–18.84)	0.00 (0.00–7.14)	6.00 (2.06–16.22)	0.00 (0.00–7.14)	0.00 (0.00–7.14)	8.00 (3.15–18.84)	2.00 (0.35–10.50)	2.00 (0.35–10.50)	6.00 (2.06–16.22)	4.00 (1.10–13.46)	2.00 (0.35–10.50)	0.00 (0.00–7.14)	2.00 (0.35–10.50)	16.00 (8.34–28.51)	8.00 (3.15–18.84)	0.00 (0.00–7.14)	0.00 (0.00–7.14)	2.00 (0.35–10.50)	0.00 (0.00–7.14)	0.00 (0.00–7.14)	2.00 (0.35–10.50)
Rottweiler	1.32 (0.23–7.08)	1.32 (0.23–7.08)	13.16 (7.31–22.55)	1.32 (0.23–7.08)	6.58 (2.84–14.49)	2.63 (0.72–9.10)	0.00 (0.00–4.81)	0.00 (0.00–4.81)	1.32 (0.23–7.08)	9.21 (4.53–17.81)	1.32 (0.23–7.08)	0.00 (0.00–4.81)	0.00 (0.00–4.81)	1.32 (0.23–7.08)	1.32 (0.23–7.08)	9.21 (4.53–17.81)	0.00 (0.00–4.81)	13.16 (7.31–22.55)	0.00 (0.00–4.81)	2.63 (0.72–9.10)	1.32 (0.23–7.08)	1.32 (0.23–7.08)	5.26 (2.07–12.77)	0.00 (0.00–4.81)	2.63 (0.72–9.10)
Shetland Sheepdog	0.00 (0.00–6.42)	0.00 (0.00–6.42)	0.00 (0.00–6.42)	0.00 (0.00–6.42)	12.50 (6.19–23.63)	12.50 (6.19–23.63)	1.79 (0.32–9.45)	0.00 (0.00–6.42)	0.00 (0.00–6.42)	1.79 (0.32–9.45)	0.00 (0.00–6.42)	1.79 (0.32–9.45)	10.71 (5.00–21.47)	3.57 (0.98–12.12)	0.00 (0.00–6.42)	0.00 (0.00–6.42)	1.79 (0.32–9.45)	12.50 (6.19–23.63)	1.79 (0.32–9.45)	1.79 (0.32–9.45)	0.00 (0.00–6.42)	1.79 (0.32–9.45)	0.00 (0.00–6.42)	1.79 (0.32–9.45)	3.57 (0.98–12.12)
Staffordshire Bull Terrier	2.82 (0.78–9.70)	1.41 (0.25–7.56)	5.63 (2.21–13.61)	8.45 (3.93–17.24)	9.86 (4.86–18.98)	0.00 (0.00–5.13)	1.41 (0.25–7.56)	0.00 (0.00–5.13)	2.82 (0.78–9.70)	2.82 (0.78–9.70)	0.00 (0.00–5.13)	0.00 (0.00–5.13)	4.23 (1.45–11.70)	2.82 (0.78–9.70)	2.82 (0.78–9.70)	1.41 (0.25–7.56)	1.41 (0.25–7.56)	18.31 (11.02–28.85)	4.23 (1.45–11.70)	2.82 (0.78–9.70)	1.41 (0.25–7.56)	2.82 (0.78–9.70)	2.82 (0.78–9.70)	2.82 (0.78–9.70)	2.82 (0.78–9.70)
Weimaraner	1.35 (0.24–7.27)	0.00 (0.00–4.94)	1.35 (0.24–7.27)	5.41 (2.12–13.09)	12.16 (6.53–21.53)	2.70 (0.74–9.33)	0.00 (0.00–4.94)	8.11 (3.77–16.58)	1.35 (0.24–7.27)	9.46 (4.66–18.26)	0.00 (0.00–4.94)	1.35 (0.24–7.27)	0.00 (0.00–4.94)	1.35 (0.24–7.27)	0.00 (0.00–4.94)	5.41 (2.12–13.09)	0.00 (0.00–4.94)	17.57 (10.56–27.77)	1.35 (0.24–7.27)	1.35 (0.24–7.27)	0.00 (0.00–4.94)	2.70 (0.74–9.33)	1.35 (0.24–7.27)	1.35 (0.24–7.27)	8.11 (3.77–16.58)
West Highland White Terrier	0.00 (0.00–3.85)	0.00 (0.00–3.85)	1.04 (0.18–5.67)	1.04 (0.18–5.67)	5.21 (2.24–11.62)	4.17 (1.63–10.23)	0.00 (0.00–3.85)	0.00 (0.00–3.85)	2.08 (0.57–7.28)	3.13 (1.07–8.79)	1.04 (0.18–5.67)	3.13 (1.07–8.79)	8.33 (4.28–15.59)	2.08 (0.57–7.28)	1.04 (0.18–5.67)	3.13 (1.07–8.79)	1.04 (0.18–5.67)	14.58 (8.89–23.00)	5.21 (2.24–11.62)	3.13 (1.07–8.79)	0.00 (0.00–3.85)	2.08 (0.57–7.28)	1.04 (0.18–5.67)	1.04 (0.18–5.67)	7.29 (3.58–14.29)
Whippet	0.00 (0.00–6.53)	0.00 (0.00–6.53)	3.64 (1.00–12.32)	1.82 (0.32–9.61)	3.64 (1.00–12.32)	3.64 (1.00–12.32)	1.82 (0.32–9.61)	1.82 (0.32–9.61)	0.00 (0.00–6.53)	0.00 (0.00–6.53)	3.64 (1.00–12.32)	0.00 (0.00–6.53)	3.64 (1.00–12.32)	1.82 (0.32–9.61)	0.00 (0.00–6.53)	7.27 (2.86–17.26)	0.00 (0.00–6.53)	12.73 (6.30–24.02)	1.82 (0.32–9.61)	0.00 (0.00–6.53)	1.82 (0.32–9.61)	0.00 (0.00–6.53)	0.00 (0.00–6.53)	5.45 (1.87–14.85)	3.64 (1.00–12.32)

**Fig. 3:**
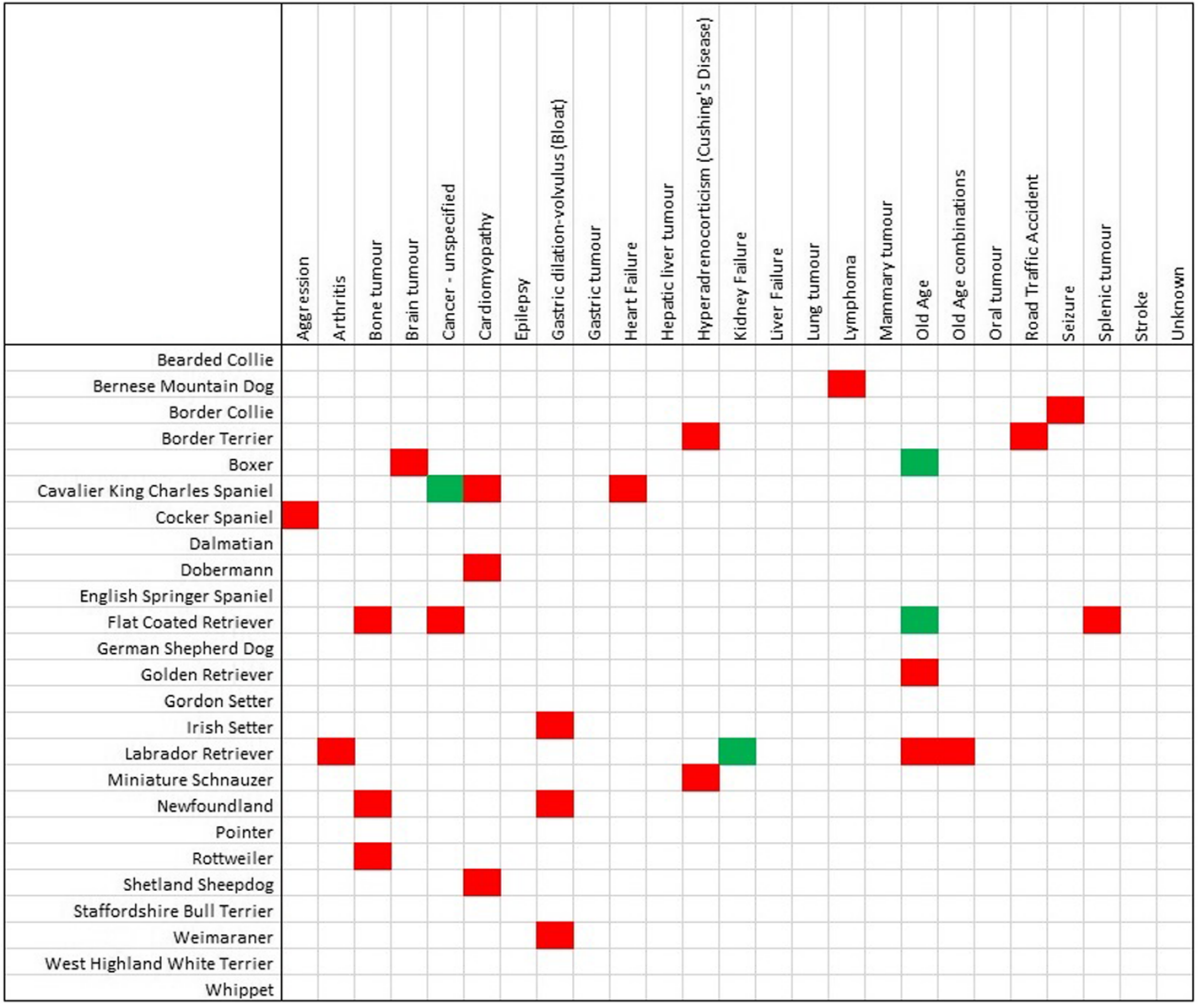
Significant differences in proportional mortality within breed compared to overall. Significantly higher (red), and lower (green) within breed proportional mortality (WBPM) than overall proportional mortality (OPM) (*P* < 0.05) for the 25 most commonly reported causes of death among the twenty-five breeds with > 50 deaths reported with ≥50 reports (*n* = 3744 deaths)

Figure 3 illustrates higher (red), and lower (green) WBPM than OPM (*P* < 0.05) for specific causes of death among the twenty-five common breeds in the study. None of the breeds showed a significant difference between WBPM and OPM for epilepsy, gastric tumour, hepatic/liver tumour, liver failure, lung tumour, mammary tumour, oral tumour, stroke and unknown. Figure 3 can be used to assess predisposition within-breeds to certain causes of death. For example, Flat Coated Retrievers had a higher WBPM of death or euthanasia due to bone tumours, unspecified cancer and splenic tumours, and a lower WBPM of death or euthanasia from old age than OPM across the whole survey. Conversely, Labrador Retrievers had a higher WBPM of death or euthanasia due to arthritis, old age and old age combinations, and a lower WBPM of death from kidney failure than OPM across the whole survey.

### Longevity by mortality

**Fig. 4:**
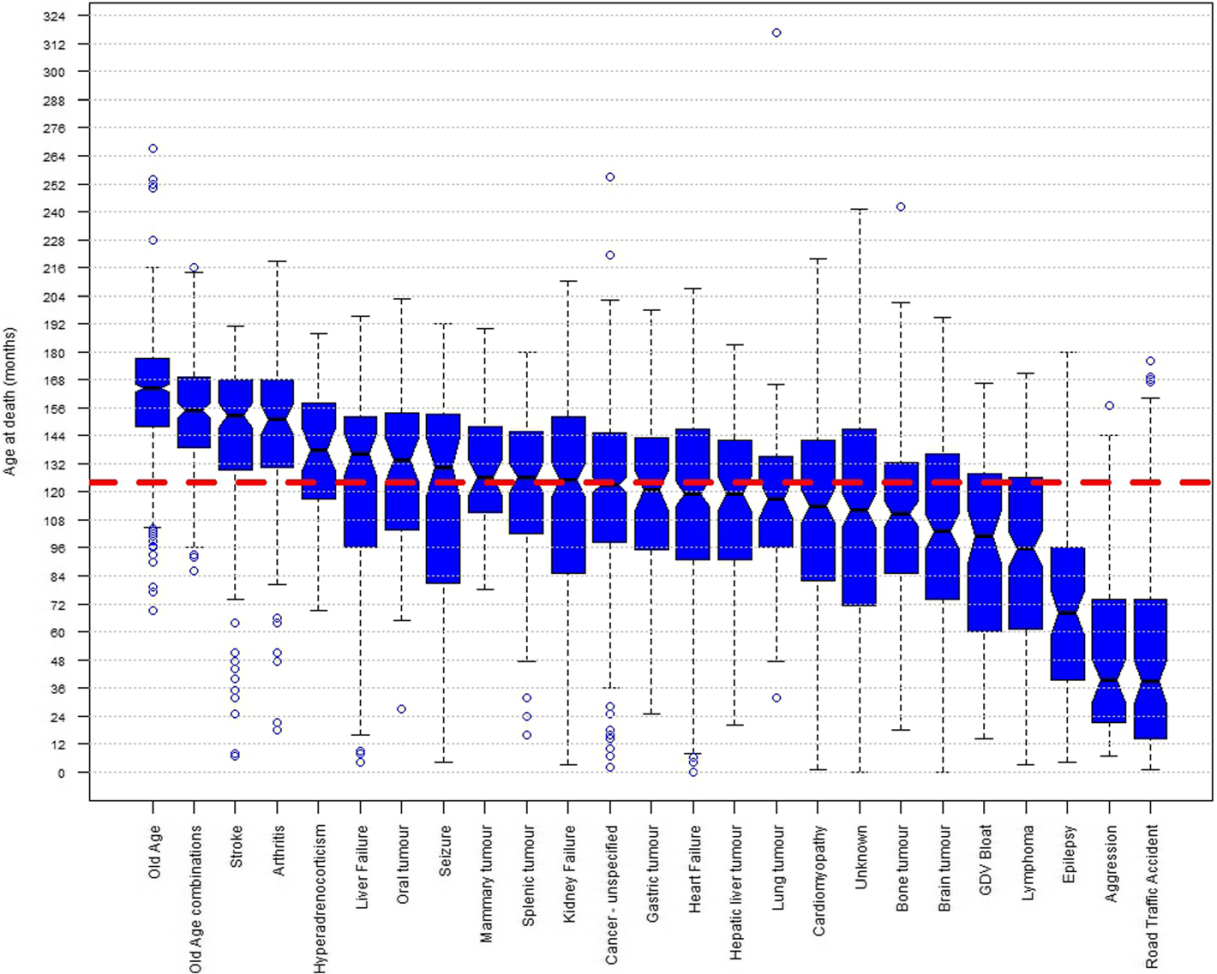
Box and Whisker plot of age at death across common causes of death. Notched Box and Whisker plot of age at death (months) for the 25 causes of death with ≥50 deaths reported. ‘Notches’ in the boxes indicate the approximate 95% confidence interval of the median. The red dashed line indicates the median age at death of all dogs in the survey (n = 5663) of 124 months, and the thickness of this line approximates to the 95% confidence interval (122.57 to 125.43 months)

The median age at death varied widely across the 25 common causes of death. Figure 4 uses a notched box and whisker plot to show variation in distribution of age at death across causes of death with ≥50 deaths reported; the ‘notches’ in the boxes indicate the approximate 95% confidence interval of the median. The cause of death with the oldest median age at death was old age (164.5 months; 13.71 years), and the cause of death with the youngest median age at death was road traffic accident (38.5 months; 3.21 years). The upper boundary of the approximate 95% confidence interval of median age at death was lower than 122.57 months (the lower boundary of the approximate 95% confidence interval of the median using all data) for nine of the twenty-five causes (cardiomyopathy, unknown, bone tumour, brain tumour, gastric dilation-volvulus [GDV or bloat], lymphoma, epilepsy, aggression and road traffic accident) identifying these disorders as tending to result in death at an earlier age. The lower boundary of the approximate 95% confidence interval of median age at death was higher than 125.43 months (the upper boundary of the approximate 95% confidence interval of the median using all data) for six of the twenty-five causes (old age, old age combinations, stroke, arthritis, hyperadrenocorticism/ Cushing’s disease and liver failure) identifying these disorders as tending to result in deaths at an older age.

## Discussion

This study of over five thousand deaths in pedigree dogs provides up-to-date information on longevity and causes of mortality in UK Kennel Club registered dogs that can support evidence-based efforts to improve health and welfare in dogs [11]. By reporting causes of death that have significantly higher and lower proportional mortality within breeds compared to all dogs, these results can support ongoing efforts to focus health reforms on priority disorders in individual breeds [28]. The results on median longevity by breed provide data that can assist current and prospective owners to manage their lifespan expectations for their dogs.

The overwhelming majority of reported deaths in dogs (79.58%) in this study involved euthanasia. However, post-mortem examinations to determine definitively the precise condition, disease or failing that led to the death were not commonly undertaken (5.56%). These findings concur with results reported from primary-care veterinary practice in the UK which reported that 86.4% of deaths involved euthanasia [7]. Although euthanasia entails an artificial foreshortening of lifespan, its time of occurrence often represents the approximate point at which the dog’s life is adjudged by the owner and the veterinarian to no longer be ‘worth’ living, either for ethical reasons pertaining to impairment of quality of the dog’s life and/or due to convenience in terms of management (of illness or behaviour) [29, 30]. The balancing of the objectives of length and quality of life has particular poignancy for euthanasia due to behavioural rather than physical health problems (notably aggression, which accounted for 85, or 1.5% of, deaths in the current study), since these undesirable behaviours may be substantially influenced by environmental influences unique to the individual, including failures in management and training or unrealistic expectations by owners on how their dog should behave [31, 32].

Death is an inevitable end to every life and therefore the emotional consequences for owners from the death of a companion animal are ultimately unavoidable and should be anticipated and prepared for [33]. Although death may be postponed at a population or individual level by improved healthcare, extended longevity by itself does not necessarily imply an improved or even a good quality of life, so a delicate balancing act exists between longevity and acceptable quality of life in order to optimise welfare in species where euthanasia is an option [34]. A shorter but largely healthy lifespan (the healthy component is sometimes called the healthspan [35]) with a rapid decline to death may offer better welfare than a longer but more ailed life with a slow and painful decline to death. This concept has been encompassed in the ‘longevity dividend’ in humans [36]. The ethical issues surrounding healthspan in dogs are exemplified by the emotional conflicts generated in owners and veterinarians during decision-making between euthanasia versus waiting for unassisted death in dogs [37, 38]. Ensuring adequate ‘quality of life’ of the animal is often the overriding priority for the owner and veterinarian, and once it is judged that the injury, disorder or general process of decline affecting the animal has taken sufficient toll on welfare, a decision may be made to foreshorten life for humane reasons [39]. Thus, a paradox may occur where an increased appreciation of welfare in older dogs may lead to greater use of euthanasia and therefore result in a shortening of general lifespan. Additionally, a generally shorter lifespan of some breeds compared to others should not be viewed as necessarily a welfare problem per se, provided there is a high quality of life during the living years and that the death process is relatively benign. Conversely, welfare concerns should be raised for breeds that die commonly from lengthy, debilitating and painful disorders, particularly when these are accompanied by a general foreshortened lifespan.

The current study reports that the median age at death across all breeds was 10.33 years. This is similar to previously reported estimates: 11.25 years in UK Kennel Club registered dogs [9]; 11.9 years (IQR 8.4 to 14.0 years) from primary veterinary practice data on UK ‘purebred’ dogs [7]; and 10 years (IQR 6 to 12 years) reported from a survey conducted by the Danish Kennel Club [10]. However, substantial variation in median longevity across breeds was also shown in the current study (Fig. 2). The longest-lived breed was the West Highland White Terrier (median age at death of 12.71 years) and the shortest was the Dobermann Pinscher (median age at death of 7.67 years). However, as discussed above, such variation in longevity is not unexpected based on previous studies that reported similar results [7–10, 18] and in part reflects the extent of phenotypic variation that exists across the spectrum of domestic dog breeds. Indeed, a general inverse correlation of longevity and body size is well established among dog breeds, with ‘giant’ breeds in general often exhibiting notably shorter life expectancy than small or miniature breeds [7–9, 40]. Several breeds in our study had median ages at death significantly lower than the overall median longevity (Flat Coated Retriever, German Shepherd Dog, Boxer, Rottweiler, Bernese Mountain Dog and Dobermann) which may raise health concerns in these breeds and prompt exploration on common causes of death in efforts to redress longevity deficits. However, as argued above, shorter life expectancy per se need not intrinsically imply welfare impairment, on the proviso that such shorter lives are generally healthy and that the dying process is not protracted or malign. Conversely, breeds determined as having a longer life expectancy should not automatically be regarded as being ‘healthier’ since it may be that such breeds are subject to long and distressing periods of decline to the point of euthanasia or death. Therefore, in order to gain greater insights into the nature of ageing, decline and ultimately death within breeds, it is necessary to consider the welfare costs of the common specific causes of death together with variation in longevity.

Substantial variation in median longevity across specific causes of death was also identified in the current study (Fig. 4). Some of these differences are intuitive; for example, the median ages at death from ‘old age’ and ‘old age combinations’ (13.71 years and 12.92 years respectively) were higher than the overall median age at death (10.33 years) as might be predicted. Specific causes of death with higher median ages at death may be described as ‘diseases of ageing’ (e.g. stroke, 12.71 years; arthritis, 12.58 years). Conversely, causes of death with lower median ages at death may be viewed as life curtailing and possibly of greater welfare impact in the sense of years of potential life lost (e.g. road traffic accident, 3.21 years; aggression, 3.25 years; epilepsy, 5.67 years) [32]. These disorder-based longevity data offer insights that can assist with disorder prioritisation for reforms within breeds that can optimise the welfare gains from the effort and resources expended.

Some significant differences were identified between the within-breed proportional mortality (WBPM) from individual disorders and the overall proportional mortality (OPM) in some breeds that can assist to identify life-limiting predispositions in these breeds (Fig. 3). At least one breed had a significantly higher or lower WBPM than OPM for 16 of the 25 common causes of death analysed. Many of these associations concur with previous reports in these breeds. For example, our study determined a higher proportional mortality from cardiac disorders in the Cavalier King Charles Spaniel (WBPM of 19.82% for heart failure and 10.81% for cardiomyopathy compared to the OPM of 4.89% and 2.93% respectively) which is concordant with previous reports that cardiac conditions were the most common cause of death in this breed [9, 41, 42]. Similarly, Flat Coated Retrievers were more likely to die from bone cancer, unspecified cancer and splenic tumours, in line with previous findings of high cancer-related morbidity and mortality in this breed [4, 43–45]. When taken with the apparently foreshortened median lifespan of Flat Coated Retrievers (9.5 years), these results imply that the breed is predisposed to these types of cancers and that this cancer predisposition likely contributes to a substantially curtailed life expectancy, which therefore may be viewed as a welfare problem. Figure 3 also revealed or confirmed other breeds at higher risk of mortality for specific conditions; for example, Irish Setter, Newfoundland and Weimaraner from GDV [46, 47], Dobermann and Cavalier King Charles Spaniel from cardiomyopathy [48, 49], Newfoundland and Rottweiler from bone cancer (in addition to the Flat Coated Retriever) [50], and Cocker Spaniel from aggression [32]. There were also some unexpected and perhaps less well established findings, such as significantly higher mortality from road traffic accidents in the Border Terrier. However, the relatively small sample sizes for some breeds and disorders mean that novel findings should be treated as hypothesis generators that are validated in later confirmatory studies [51].

**Fig. 5:**
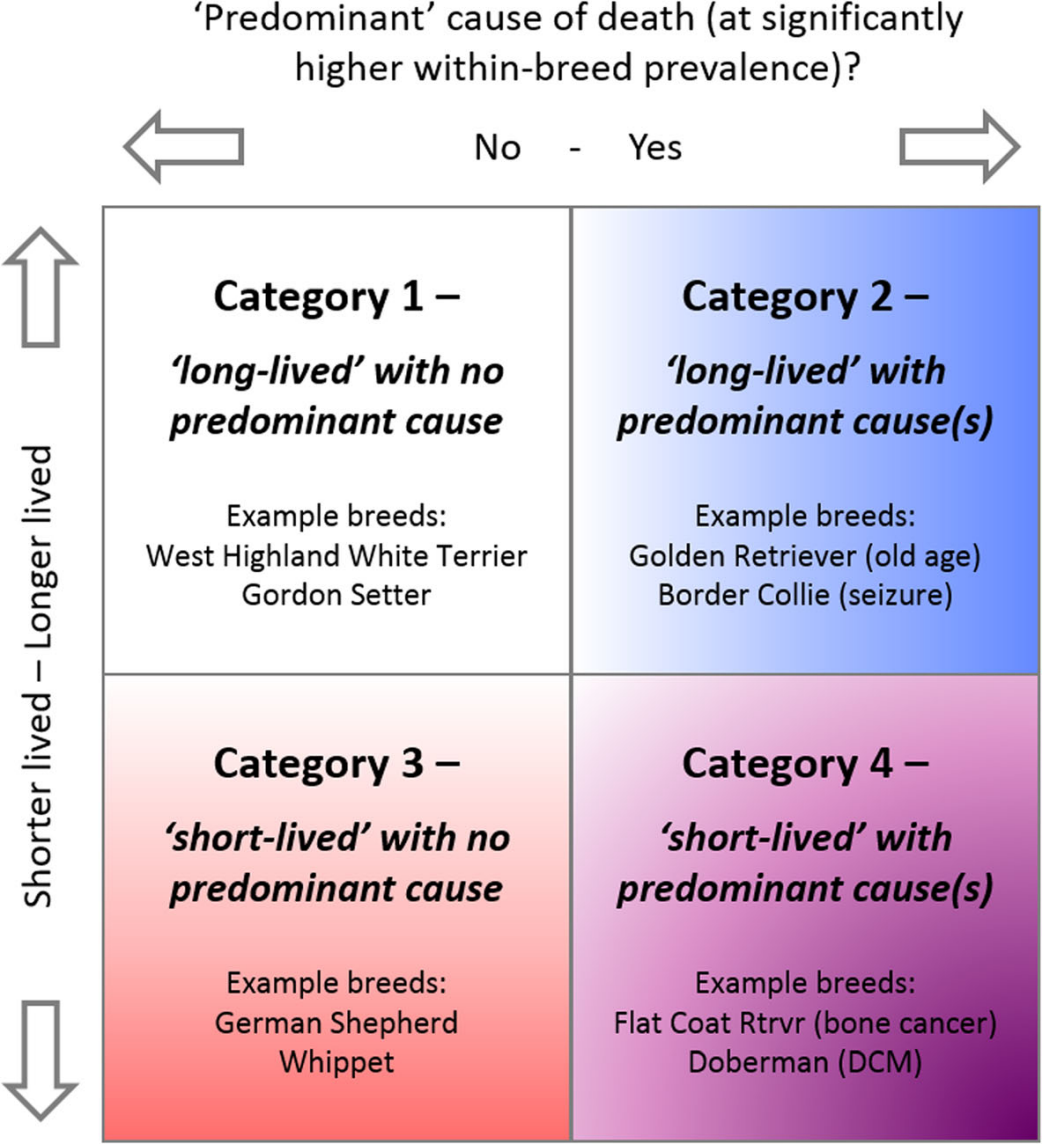
Diagrammatic representation of the 4 categories breeds may be assigned to based on longevity and high within breed prevalence of particular cause(s) of death. Longer and shorter lived (red) shown on vertical; binary category on horizontal indicating no cause of death at higher within breed prevalence (left) and one or more causes at higher within breed prevalence (right)

Merging the results of longevity by breed with WBPM of causes of death by breed may allow assignment of breeds to one of four categories: 1) long-lived with no specific cause of death at a raised proportional mortality (e.g. West Highland White Terrier, Bearded Collie, Gordon Setter); 2) long-lived with at least one cause of death at higher proportional mortality (Labrador [old age, old age combinations and arthritis], Golden Retrievers [old age], Border Collie [seizure]); 3)

short-lived with no cause of death at a raised proportional mortality (German Shepherd Dog, Whippet); and 4) short-lived with at least one cause of death at a higher proportional mortality (Dobermann [cardiomyopathy], Bernese Mountain Dog [lymphoma], Flat Coated Retriever, Rottweiler and Newfoundland [bone cancer], Cavalier King Charles Spaniel [cardiomyopathy and heart failure]). This is shown visually in Fig. 5. Category 4 (short-lived with increased probability of at least one cause of death) could be considered to represent breed predisposition for serious life-limiting conditions and to represent potential welfare concerns, particularly where the cause of death at a raised proportional mortality has a low median age (e.g. lymphoma [7.96 years]; bone tumour [9.21 years]). Conversely categories 1 (long lived with no raised proportional mortality of a cause of death) and 3 (short lived with no raised proportional mortality of a cause of death) may represent a general variation in longevity associated with factors that apply across all dogs, such as body size, but with no obvious disease or disorder as an accentuating driver for death [7, 9].

The causes of death reported in this study with the highest proportional mortality across all breeds were old age (13.77%), unspecified cancer (8.69%) and heart failure (4.89%). The proportional mortality due to old age was lower than that reported in a survey conducted by the Danish Kennel Club (20.8%) [10] and an earlier UK study comprising UK Kennel Club registered dogs (17.8%) [9]. The Danish study also reported a higher frequency cause of death due to cancer (14.5%) than the current study [10]. Differing reported proportional mortality values between studies may reflect differences in disease classification; for example, cancer was often listed as the specific variant in the current study rather than as a general ‘cancer’ category [6]. Additionally, the current study only classified ‘old age’ as the cause of mortality when ‘age’ or ‘old age’ was solely cited by the respondent; when multiple disorders/conditions were listed along with ‘old age’ this was recorded as ‘old age combinations’; adding the proportional mortality from ‘old age’ and ‘old age combinations’ in our study identifies that 17.8% of dogs died from causes relating to old age. This implies that just under a fifth of dogs in this survey died from conditions that the owner considered to be intrinsically linked to ageing and decline. The rationale for including ‘old age combinations’ as a cause of death was to remove the subset of the specific causes of death from the overall analyses that were considered by the owner to reflect terminal decline rather than an intrinsic pathology. For example, ‘incontinence’, arthritis’ and ‘weak hind legs / collapse’ as causes of death imply different aetiology in ‘old’ dogs (where they are consistent with age related decline) than in dogs in their prime of life (when they would be indicative of disease or trauma).

This study had some limitations. The nested mortality survey had low uptake compared with the originating morbidity survey; only 5663 deaths were reported compared with responses relating to 43,005 live dogs in the morbidity survey [4]. An earlier survey on pedigree dogs in the UK was more successful and gathered data on 15,881 deaths [9]. The reasons behind the current low response rate are unknown but may have introduced some response rate bias [52]. Changes to breed popularity over time are likely to also introduce some temporal biases to the results [53, 54]. Breeds that are increasing in popularity are likely to have a proportionally greater number of deaths of younger dogs and therefore to shift the median longevity downwards while breeds that are decreasing in popularity will conversely show an apparent extension in longevity compared with the true situation because there are relatively more of the older dogs available to die [55, 56]. The popularity of breeds registered with the Kennel Club over the last decade is known to be non-static so this may have biased the longevity estimates downwards for breeds with increasing registrations (e.g. Whippet, Cocker Spaniel and Miniature Schnauzer) and biased the longevity estimates upwards for breeds with decreasing registrations (e.g. West Highland White Terrier, Cavalier King Charles Spaniel and Irish Setter) [57]. However, the representation of the 25 breeds with 50 or more deaths reported was broadly in line with registered population size; the correlation of number of deaths reported and total registrations of dogs born between 2000 and 2004 (the distribution of year of birth was approximately normal with averages at 2000/2001, not shown) was 0.9. Further temporal biases or influences on longevity include technological advances and improvements in veterinary treatment and care, selection occurring against disease across the time frame that coincides with the lifespan of dogs in this study, and possibly other changes in the genetics of breed populations. The biological causes for seemingly arbitrary variation in life expectancy (i.e. where there is no obvious causal disorder) across breeds (or other categories, such as sex) are unknown and likely to be hugely complex. Exploring the full depth of these complexities was outside the aims of the current study but data gathered here and from similar studies may help to reveal new insights into dog life expectancy in future analyses.

## Conclusion

Substantial variation in the median lifespan and the prominent causes of death exists across breeds. This study has identified individual breeds that have both a low median lifespan and also a high proportional mortality for one or more specific causes of death. Breeds with this combination are highlighted with potential welfare concerns that may need to be addressed.

## Additional file

Additional file 1: Number of reported deaths (and percentage of total reported) per breed. Number of reported deaths (and percentage [prevalence, and 95% confidence interval]) per cause. (XLSX 24 kb)

## Abbreviations

95% CI: 95% confidence interval
GDV: Gastric dilation volvulus
IQR: Interquartile range
OPM: Overall proportional mortality
WBPM: Within breed proportional mortality

## Footnotes

1 Refers to the breeds recognised at the time of the survey. Six breeds have been recognised by The Kennel Club since the survey closed**.**
